# Cost and Impact of Voluntary Medical Male Circumcision in South Africa: Focusing the Program on Specific Age Groups and Provinces

**DOI:** 10.1371/journal.pone.0157071

**Published:** 2016-07-13

**Authors:** Katharine Kripke, Ping-An Chen, Andrea Vazzano, Ananthy Thambinayagam, Yogan Pillay, Dayanund Loykissoonlal, Collen Bonnecwe, Peter Barron, Eva Kiwango, Delivette Castor, Emmanuel Njeuhmeli

**Affiliations:** 1 Health Policy Project, Avenir Health, Washington, District of Columbia, United States of America; 2 Health Policy Project, Futures Group, Washington, District of Columbia, United States of America; 3 U.S. Agency for International Development, Pretoria, South Africa; 4 National Department of Health, Pretoria, South Africa; 5 School of Public health, University of the Witwatersrand, Johannesburg, South Africa; 6 Joint United Nations Programme on HIV/AIDS, Pretoria, South Africa; 7 U.S. Office of the Global AIDS Coordinator, Washington, District of Columbia, United States of America; 8 U.S. Agency for International Development, Washington, District of Columbia, United States of America; University of Ottawa, CANADA

## Abstract

**Background:**

In 2012, South Africa set a goal of circumcising 4.3 million men ages 15–49 by 2016. By the end of March 2014, 1.9 million men had received voluntary medical male circumcision (VMMC). In an effort to accelerate progress, South Africa undertook a modeling exercise to determine whether circumcising specific client age groups or geographic locations would be particularly impactful or cost-effective. Results will inform South Africa’s efforts to develop a national strategy and operational plan for VMMC.

**Methods and Findings:**

The study team populated the Decision Makers’ Program Planning Tool, Version 2.0 (DMPPT 2.0) with HIV incidence projections from the Spectrum/AIDS Impact Module (AIM), as well as national and provincial population and HIV prevalence estimates. We derived baseline circumcision rates from the 2012 South African National HIV Prevalence, Incidence and Behaviour Survey. The model showed that circumcising men ages 20–34 offers the most immediate impact on HIV incidence and requires the fewest circumcisions per HIV infection averted. The greatest impact over a 15-year period is achieved by circumcising men ages 15–24. When the model assumes a unit cost increase with client age, men ages 15–29 emerge as the most cost-effective group. When we assume a constant cost for all ages, the most cost-effective age range is 15–34 years. Geographically, the program is cost saving in all provinces; differences in the VMMC program’s cost-effectiveness across provinces were obscured by uncertainty in HIV incidence projections.

**Conclusion:**

The VMMC program’s impact and cost-effectiveness vary by age-targeting strategy. A strategy focusing on men ages 15–34 will maximize program benefits. However, because clients older than 25 access VMMC services at low rates, South Africa could consider promoting demand among men ages 25–34, without denying services to those in other age groups. Uncertainty in the provincial estimates makes them insufficient to support geographic targeting.

## Introduction

In 2005, researchers in South Africa published results from the first randomized controlled trial demonstrating the effectiveness of voluntary medical male circumcision (VMMC) in preventing HIV infection [1]. These findings, along with those from subsequent trials in Uganda and Kenya, provided definitive evidence that VMMC reduces the risk of female-to-male HIV transmission by approximately 60% [2,3]. The World Health Organization (WHO) and the Joint United Nations Programme on HIV/AIDS (UNAIDS) embraced VMMC as an effective intervention for HIV prevention in 2007, recommending that VMMC be promoted as part of a comprehensive HIV prevention package in countries with high HIV prevalence and low prevalence of male circumcision (MC; includes traditional circumcisions) [4]. The group identified 13 countries, including South Africa, where accelerated scale-up to 80% coverage of MC among males ages 15–49 should be prioritized [4].

HIV prevalence in South Africa is estimated at 12.2% (all ages), and fewer than half (46.4%) of men ages 15–49 report being circumcised [5]. South Africa has populations in which circumcision is culturally normative. Circumcision is seasonal: most procedures are performed in winter. Both MC and HIV prevalence vary by age and province, at times in an inverse relationship. For example, Kwa-Zulu Natal has the highest HIV prevalence in the country (16.9%) but the second-lowest percentage of circumcised adult males (23.2%).

Modeling conducted in 2011 projected that by scaling up VMMC coverage to 80% among males ages 15–49, the country could avert more than 1 million HIV infections over 15 years [6]. In response, the current Strategic Plan for the Scale up of Medical Male Circumcision (MMC) in South Africa aims to circumcise 4.3 million men ages 15–49 by 2016 [7]: the highest VMMC target among all 13 priority countries [6]. By the end of the South African Government’s fiscal year (FY) 2014/15, ending in March 2015, the country had conducted only 1.9 million VMMCs among this age group, making it unlikely to reach its VMMC targets by the end of 2016. VMMCs conducted to date are projected to avert 94,000 infections by 2025 [8].

To ensure accelerated progress of the campaign, the country must consider program targets and priorities in the context of trends in HIV prevalence, MC prevalence, program implementation, and uptake. Client age data for 2010–2014 from the U.S. President’s Emergency Plan for AIDS Relief (PEPFAR) show that service uptake varies according to age group, with greater than 60% of VMMCs performed among young men ages 10–19 (S1 Table). Men ages 10–19 years are accessing services more frequently than expected given population estimates, while clients older than 25, and especially older than 35, access VMMC services less frequently than expected [9,10]. Importantly, the country’s 4.3 million target and the program data at government-only sites do not take into account clients ages 10–14 years, though this group made up more than 40% of the client base in 2014, according to PEPFAR program data. When the 10- to 14-year-olds were included, the total number of circumcisions conducted in South Africa was estimated to be nearly 2.7 million by the end of FY 2014/15 (S1 Table), suggesting that both the targets and the reporting by age group may need to be reconsidered.

Geographic variability is another important factor in program planning and evaluation. The South Africa VMMC program was first piloted in KwaZulu-Natal in 2010, followed by rollout to all other provinces the following year. Resources for VMMC were allocated among the provinces based on population size and epidemiology (HIV and MC prevalence). For the years 2010–2014, the greatest numbers of VMMCs were performed in KwaZulu-Natal (636,041) and Gauteng (412,952) (S2 Table). Notably, some provinces demonstrated faster scale-up. For example, in FY 2011/12, Mpumalanga province reported performing 14,029 male circumcisions; by the end of FY 2014/15, the total number completed reached 186,414.

In the context of these trends, policymakers in South Africa sought additional data to help them to reassess national VMMC targets and develop a more strategic approach to program implementation. Because the model that was applied in 2011 [6] was not able to disaggregate target populations by age or province, the authors applied a new model—the Decision Makers’ Program Planning Tool, Version 2.0 (DMPPT 2.0)—to examine the cost and impact of scaling up circumcision among various age groups and provinces. Results from this new model will inform South Africa’s efforts to develop a national strategy and operational plan for VMMC.

## Methods

### Ethical Considerations

No personal or medical data were used in this study; therefore no ethical approval was required. All data populating the model were derived from publicly available sources, with the exception of the unit costs, the sources of which are described below. All data were collected prior to the study initiation; authors were not involved in collection of the data. No individual research participants or personal data were involved with this modeling study; as such, there was no required consent form. No waiver of ethical approval was obtained for this study.

### DMPPT 2.0 Model

The DMPPT 2.0 model is described in detail in [11]. Briefly, DMPPT 2.0 is a simple compartmental model implemented in Microsoft Excel 2010, designed to analyze the effects of age at circumcision on program impact and cost. The DMPPT 2.0 model tracks the number of circumcised males in newborns and in each five-year age group over time, taking into account age progression and mortality. The model calculates discounted VMMC program costs and HIV infections averted in the population each year in a user-specified VMMC scale-up strategy, compared with a baseline scenario in which the MC prevalence remains the same as it was. The baseline scenario assumes that traditional or other circumcisions that produced the baseline MC prevalence continue at the same rate as before the VMMC program was initiated.

### South Africa Data Sources

A national DMPPT 2.0 model was created, in addition to a separate model for each of the nine provinces in South Africa (Eastern Cape, Free State, Gauteng, KwaZulu-Natal, Limpopo, Mpumalanga, Northern Cape, North West, and Western Cape). All model inputs are available in the supplemental materials (S1 and S2 Appendices) and described here. The DMPPT 2.0 model is populated with population, mortality, and HIV incidence and prevalence projections from an external source. For the South Africa country application, we used the Spectrum/AIM model [12], which projects population size, mortality, and HIV prevalence and incidence based on data empirically collected from the country. The national DMPPT 2.0 model was populated from the validated national Spectrum/AIM file obtained from UNAIDS on January 13, 2014, entitled “South Africa_HSRC July 26 2013.” Validated provincial Spectrum/AIM files created by the country were obtained with permission on December 23, 2013; these were used to populate the provincial DMPPT 2.0 models.

The MC prevalence by age group in the model base year (2014) was from the South African National HIV Prevalence, Incidence and Behaviour Survey, 2012 [5]. The unit cost of VMMC used in the analysis was $125 USD, as estimated by local stakeholders based roughly on an expenditure analysis conducted by the PEPFAR team in South Africa in 2013. (All subsequent references to currency are in U.S. dollars, and all costs are economic costs from the funder perspective, including above-facility-level costs.) For antiretroviral therapy (ART), we used a cost per patient year of treatment of $377 from the National AIDS Cost Model, provided on February 17, 2014 by Gesine Meyer-Rath (a specialist in the economics of HIV and ART in resource-limited settings at the Health Economics and Epidemiology Research Office, University of Witwatersrand, South Africa). ART costs included the total cost of ART provision, including personnel, facility costs, and above-facility-level costs.

### Analytical Approaches

To examine the effect of client age on the impact of scaling up VMMC, we identified individual five-year age groups and several combined age groups, such as 10–34 or 15–29. For each individual or combined age group, we created a scenario that assumed a target of 80% MC coverage for the age group in question and left the target for the other age groups at the same level as the baseline. Each scenario scaled up MC coverage between 2014 and 2018, by applying a linear interpolation to the baseline MC prevalence for each age group in 2013 and the target coverage in 2018. After 2018, the coverage for each age group was maintained at the target level. For each scenario, we compiled the decrease in HIV incidence in the scale-up scenario compared with the baseline scenario in each year of the model, and the total number of circumcisions required during the scale-up phase (2014–2018). The following model outputs for each scenario were measured over the 15-year period between 2014 and 2028, inclusive: the total number of HIV infections averted in the population (including secondary infections averted among females; see above); the number of VMMCs per HIV infection averted; the total cost of the VMMC program; the total number of HIV infections averted; and the total cost of the VMMC program. Costs, numbers of circumcisions, and infections averted were all discounted at a rate of 5% per year [13]. Uncertainty around the age distribution of HIV incidence was calculated as described in [11].

To examine the differences in impact and cost-effectiveness across South Africa’s provinces, we compiled the cost per HIV infection averted over the 15-year period 2014–2018, inclusive, from each provincial DMMPT 2.0 file. The VMMC scale-up scenario used for this analysis was to scale up MC coverage to 80% among males ages 10–34. Uncertainty around the provincial estimates was calculated as follows: The Spectrum/AIM Uncertainty Analysis tool was run on each individual provincial Spectrum/AIM file described above. The median, lower 2.5%, and upper 97.5% bounds around the adult (ages 15–49) HIV incidence for the year 2020 were extracted from the Uncertainty tool. DMPPT 2.0 files for each region were created representing the lower and upper bounds of the HIV incidence, by multiplying the incidence in the original file by the ratio of the lower to median or upper to median values. Cost per HIV infection averted extracted from the lower bound file for each region was used as the upper bound in the analysis, and conversely for the lower bound. The lower bound of the HIV treatment cost averted was extracted from the lower bound HIV incidence file and vice versa. (Lower HIV incidence leads to higher VMMC cost per HIV infection averted and a lower treatment cost averted.)

## Results

### Age Analyses

The paper in this collection on methods [11] describes a framework comprising four metrics for assessing the impact and efficiency of VMMC using this model approach: the number of VMMCs per HIV infection averted (over 15 years), immediacy of impact (reduction in HIV incidence over five years), magnitude of impact (reduction in HIV incidence over 15 years), and cost-effectiveness (cost per HIV infection averted, over 15 years).

The methods article in this collection [11] shows that between 2014 and 2028, the number of VMMCs per HIV infection averted (VMMC/IA) in South Africa is lowest for males circumcised between the ages of 20–24 (mean: 16, uncertainty: +/- 1.6), 25–29 (mean: 15, uncertainty: +/- 1.7), and 30–34 (mean: 17, uncertainty: +/- 2.0), indicating that focusing on men in these age groups is most efficient.

**Fig 1:**
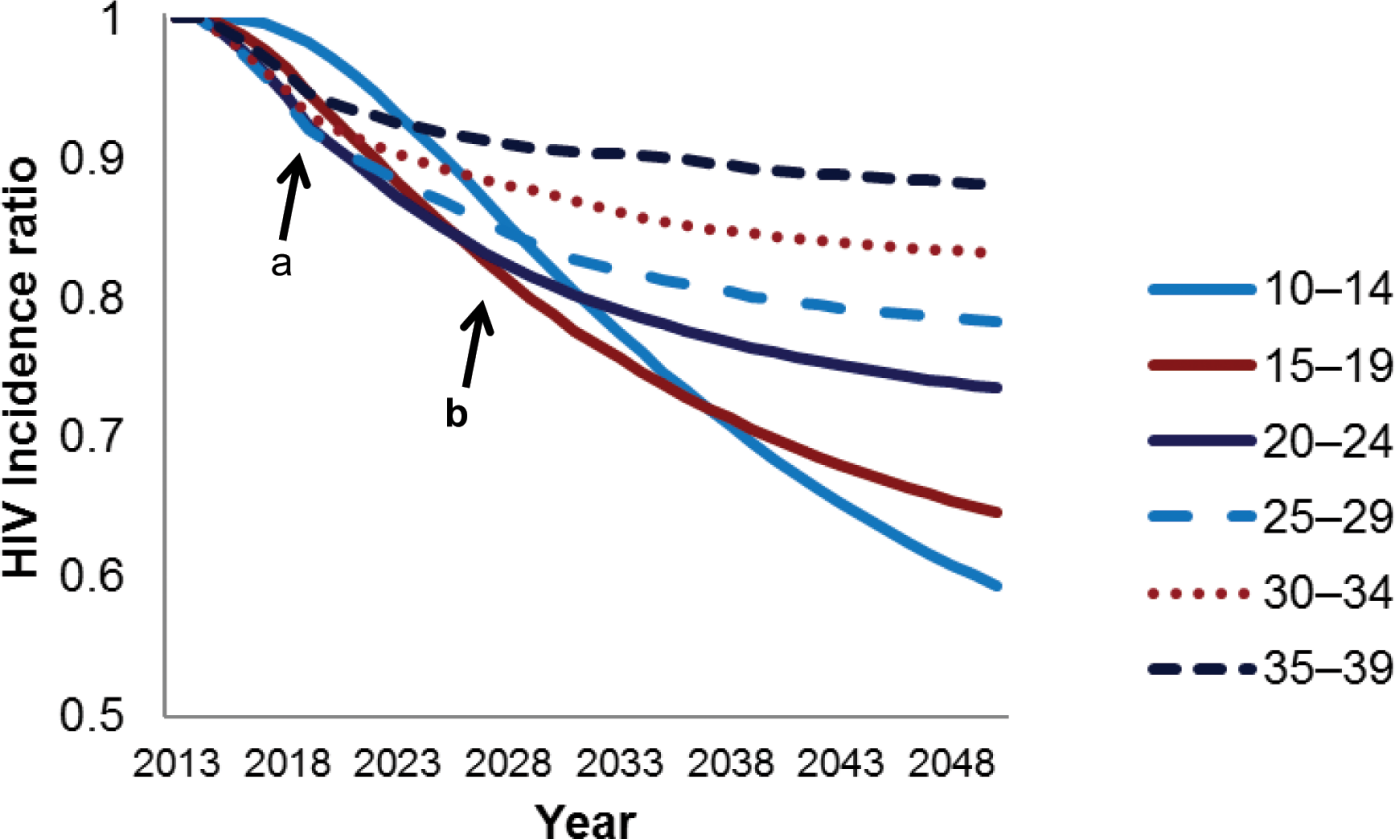
Reduction in HIV incidence with provision of VMMC to males, by age group, 2014–2050. The HIV incidence ratio represents the incidence in the scale-up scenario divided by the incidence in a population where circumcision is not scaled up over baseline levels. HIV incidence is in the entire population—males and females. Each line represents the HIV incidence ratio under a scenario in which only the indicated five-year age group is circumcised. Marker a represents a five-year period from the base year (2014). Marker b represents a 15-year period from the base year.

The impact of the VMMC program on HIV incidence rates in the short term (i.e., immediacy of impact) is presented in Fig 1, marker a. The greatest relative reduction in HIV incidence over five years is achieved by scaling up to 80% coverage among males ages 20–24, 25–29, and 30–34. The greatest magnitude of impact, defined as the greatest relative reduction in HIV incidence over 15 years, is achieved when circumcising 80 percent of the 15- to 19-year-olds and the 20- to 24-year-olds (Fig 1, marker b).

**Fig 2:**
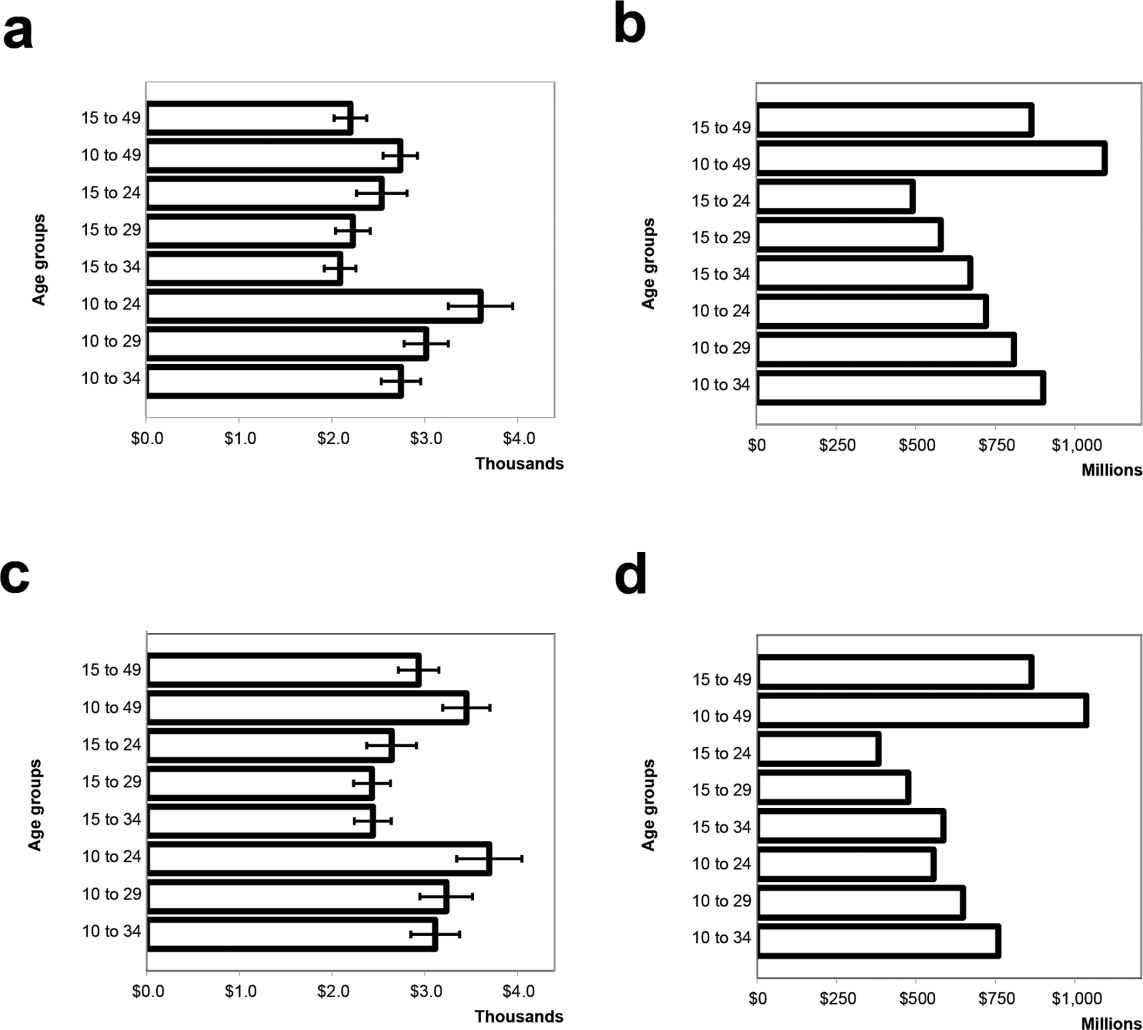
VMMC cost-effectiveness and program cost with and without increasing unit cost with client age. (a) discounted cost per HIV infection averted, 2014–2028, scenario 1: unit costs the same across all age groups; (b) discounted total cost of VMMC program, 2014–2028, scenario 1: unit costs the same across all age groups; (c) discounted cost per HIV infection averted, 2014–2028, scenario 2: increasing unit cost with increasing client age; (d) discounted total cost of VMMC program, 2014–2028, scenario 2: increasing unit cost with increasing client age. Each bar represents scale-up of VMMC among the indicated age group, compared with a reference case in which male circumcision prevalence is maintained at base levels from before the initiation of the VMMC program. Error bars represent uncertainty bounds as described in [11].

The analyses presented in Fig 1 examined fictitious scenarios in which only a single five-year age group at a time was circumcised. To assess the impact and cost-effectiveness of circumcising age groups that might be closer to actual implementation strategies, we examined several scenarios with 80% targets for combined age groups, such as 10–34 or 15–29. Among combined age groups, scaling up VMMC among the entire population ages 10–49 provides the greatest magnitude of impact (377,000 HIV infections averted [HIA]). If a narrower age group is needed for programmatic reasons, scaling up VMMC among clients ages 10–34 is still projected to avert 310,000 HIV infections, which is 84% of the HIA from scaling up VMMC among clients ages 15–49—the current strategy in South Africa (Table 1). Focusing on ages 15–34 is the most cost-effective strategy over 15 years (lowest cost per HIV infection averted), if we assume that cost does not vary by age of client (Fig 2, panel 1).

**Table 1:** HIV Infections Averted (2014–2028) in Scenarios Scaling Up VMMC among Indicated Age Groups.

Age Group	Total HIA, Thousands	UI for Total HIA	Percentage of HIA among Males and Females, 15–49	Male HIA, Thousands	UI for Male HIA	Female HIA, Thousands	UI for Female HIA
15–49 (current strategy)	370	(341, 400)	100%	248	(228, 268)	122	(112, 132)
10–49	377	(351, 403)	102%	252	(235, 270)	124	(116, 133)
15–24	183	(173, 192)	49%	122	(116, 129)	60	(57, 63)
15–29	245	(232, 259)	66%	164	(155, 174)	81	(77, 85)
15–34	303	(283, 323)	82%	203	(190, 217)	100	(93, 107)
10–24	189	(180, 198)	51%	127	(120, 133)	62	(59, 65)
10–29	253	(239, 267)	68%	169	(160, 179)	83	(79, 88)
10–34	310	(290, 330)	84%	207	(194, 221)	102	(96, 109)

HIA: HIV infections averted. UI: uncertainty interval. Each row represents scale-up of VMMC among the indicated age group, compared with a reference case in which male circumcision prevalence is maintained at base levels from before the initiation of the VMMC program. Percentage of HIA compares the HIA achieved by scale-up of VMMC in the indicated age group with the HIA achieved by scaling up among ages 15–49, the current strategy at the time the analysis was conducted. Uncertainty intervals are described in [11].

Table 2 summarizes the analyses described above, showing the priority age groups for each of the metrics in the analytical framework, according to the model. The table also displays the number of circumcisions needed required by 2018 to achieve 80 percent VMMC coverage within the corresponding age group.

**Table 2:** Priority Age Groups for Each Model Parameter within the Analytical Framework.

Parameter	Priority Age Group	Number of VMMCs Required to Reach 80% Coverage by 2018 (in Millions)
VMMC/IA	20–34	2.7
Immediacy of impact	20–34	2.7
Magnitude of impact	15–24	2.4
Cost-effectiveness	15–34	4.0

### Varying VMMC Unit Cost by Age of Client

The initial analyses of cost-effectiveness by age assumed that the VMMC unit cost did not vary by the age of the client. VMMC implementers indicated that it was more difficult to recruit older clients, so these clients would require increased targeted demand creation and special services tailored to their needs, such as lower provider-to-client ratio, and evening and weekend clinic hours. We were unable to obtain data about specifically how much more it would cost to recruit and provide services to older clients, so we conducted a sensitivity analysis using an extreme scenario, in which the cost of providing services to clients older than 40 was twice as much as providing services to clients ages 10–19, with gradual increases in the intervening age groups (Table 3**).** The DMPPT 2.0 model enables the user to specify varying costs of service delivery and demand creation by five-year client age group. The costs listed in Table 3 were entered in the model, and the scenario detailed in Table 3 was compared with the base scenario of a fixed VMMC unit cost of $125 regardless of client age.

**Table 3:** Unit Costs of VMMC by Age Group, for Cost-Sensitivity Analysis.

Age Group	Cost per Procedure
10–14	$125
15–19	$125
20–24	$150
25–29	$175
30–34	$200
35–39	$225
40–44	$250
45–49	$250
50–54	$250
55–59	$250

Demand creation costs are included.

Fig 2 shows that the cost scenario outlined in Table 3 resulted in only small changes in the most cost-effective age groups receiving VMMC compared with the base scenario. When costs increase with client age, 15- to 29-year-olds are the most cost-effective age group; 15- to 34-year-olds are most cost-effective in the base scenario of constant cost with client age. However, the uncertainty bounds around the cost-effectiveness estimates largely overlap for the different age groups in each scenario, so these differences may not be real.

### Provincial Analyses

The DMPPT 2.0 projections assessed variations in VMMC scale-up and cost-effectiveness across nine provinces in South Africa. The cost per HIV infection averted is a function of the number of VMMC/IA, which is roughly inversely related to the HIV incidence (S1 Fig**)**.

**Fig 3:**
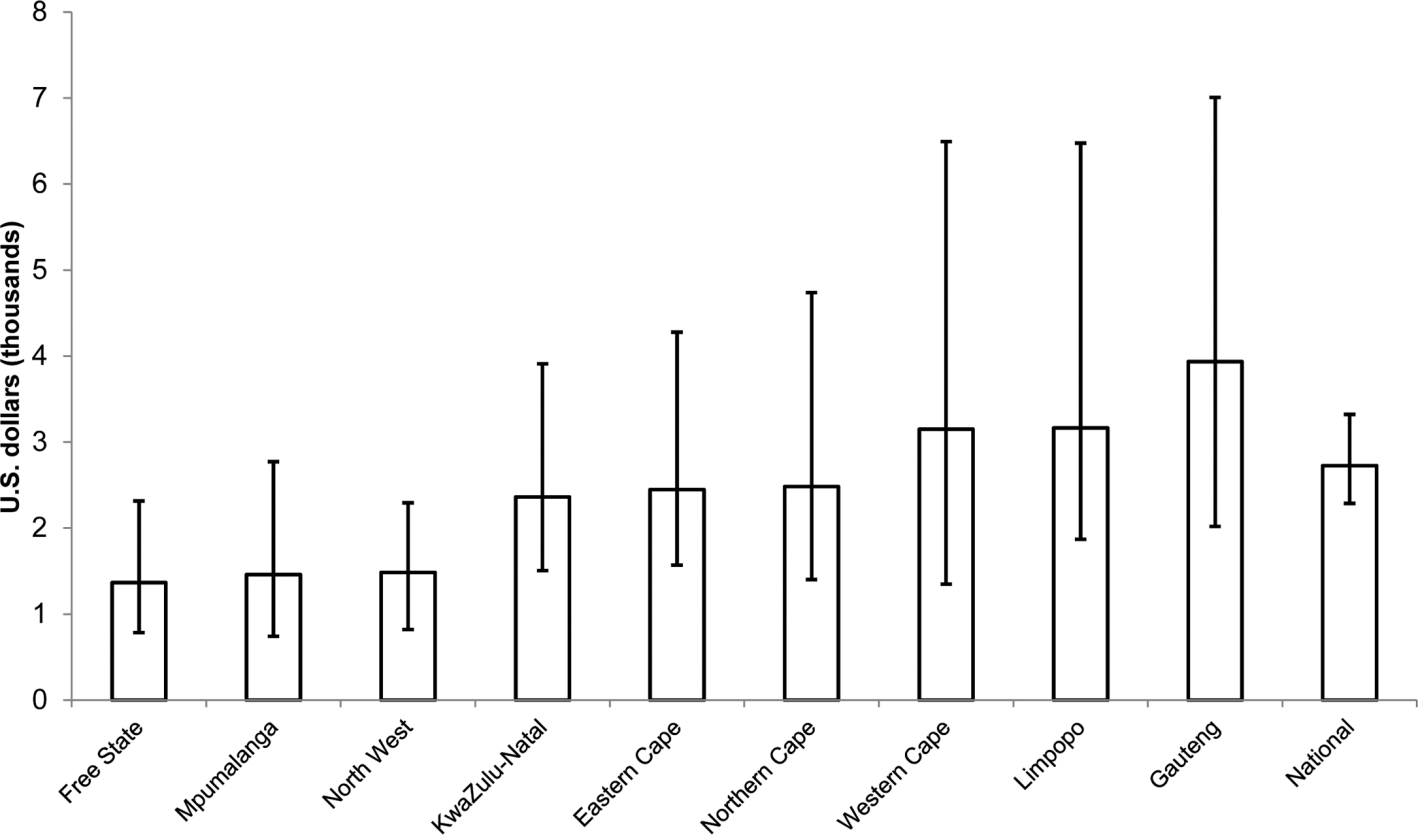
Discounted cost per HIV infection averted across provinces, 2013–2028. Each bar represents scale-up of VMMC among males ages 10–34 in the indicated province, compared with a reference case in which male circumcision prevalence is maintained at base levels from before the initiation of the VMMC program. Error bars represent uncertainty bounds as described in the Methods section.

Fig 3 shows about a threefold variation in cost per HIV infection averted across provinces. Because the provincial HIV incidence projections have wide UIs, the UIs around the cost per HIV infection averted largely overlap, so confidence in the ranking of the provinces by cost-effectiveness is low.

We also compared the cost of scaling up VMMC in each province with cost savings from avoidance of ART as a result of HIV infections averted by the VMMC program (Table 4). When the VMMC program costs less than the treatment costs averted, the program is considered cost-saving. We examined VMMC program cost savings in each program, using VMMC unit costs ranging from $125 to $225. In all provinces except Gauteng, the VMMC program was cost-saving compared with the treatment costs averted, even with a unit cost up to $225. Gauteng province was cost-saving with a unit cost of $125, but not with a unit cost of $225. Whether scaling up VMMC is cost saving depends not only on the relative cost of VMMC to ART, but also on the future HIV incidence projection, which is uncertain. The uncertainty in the HIV incidence projection for the three provinces with the lowest projected HIV incidence—Western Cape, Limpopo, and Gauteng—leads to uncertainty intervals for HIV treatment cost savings that include zero at a VMMC unit cost of $125. At a VMMC unit cost of $225, the uncertainty interval includes zero for the national projection and all provinces except Free State, Mpumalanga, and Northwest.

**Table 4:** Cost Savings of the VMMC Program in Each Province, Millions, USD, (2014–2028).

Province	Discounted Treatment Costs Averted	Discounted VMMC Program Cost with $125 unit Cost	Cost Savings with $125 Unit Cost	Discounted VMMC Program Cost with $225 Unit Cost	Cost Savings with $225 Unit Cost
Free State	$189 ($112, $329)	$50	$139 ($62, $279)	$90	$99 ($22, $239)
Mpumalanga	$258 ($136, $508)	$70	$188 ($66, $438)	$126	$132 ($10, $382)
North West	$210 ($135, $379)	$65	$145 ($70, $314)	$116	$93 ($19, $263)
KwaZulu-Natal	$623 ($376, $977)	$277	$346 ($99, $700)	$499	$124 (-$123, $478)
Eastern Cape	$226 ($129, $353)	$97	$129 ($32, $256)	$174	$52 (-$45, $179)
Northern Cape	$96 ($50, $169)	$40	$55 ($10, $129)	$72	$23 (-$22, $97)
Western Cape	$148 ($72, $345)	$77	$71 (-$5, $268)	$138	$10 (-$66, $207)
Limpopo	$150 ($73, $153)	$76	$73 (-$3, $77)	$137	$12 (-$64, $16)
Gauteng	$160 ($90, $311)	$107	$53 (-$17, $204)	$193	-$33 (-$103, $118)
National	$1,734 ($1,422, $2,066)	$841	$893 ($581, $1,225)	$1,728	$6 (-$306, $338)

Numbers in parentheses represent uncertainty bounds as described in the Methods section.

## Discussion

This DMPPT2 modeling exercise was conducted to examine the potential cost and impact of scaling up VMMC among various age groups and provinces in South Africa. Results demonstrate that the impact and cost-effectiveness vary by age-targeting strategy. The geographic prioritization is uncertain, due to the uncertainty in the provincial HIV incidence projections. Circumcising men ages 20–34 will offer the most immediate impact on HIV incidence and require the fewest circumcisions to avert one HIV infection. Over a 15-year period, the greatest impact and cost-effectiveness can be achieved by circumcising men ages 15–24 and 15–34, respectively.

The sensitivity analysis outlined in Table 3 sought to further examine cost-effectiveness in light of the increased resources required to recruit and tailor services to older clients. Even in an extreme scenario, in which VMMC service costs increase twofold for clients over 40, the analysis resulted in only small changes in the most cost-effective age groups receiving VMMC in comparison with South Africa’s current strategy (15–49 year olds). In the scenario where costs increase with client age, 15- to 29-year-olds emerge as the most cost-effective age group—a group included in the 15–34 age range shown to be the most cost-effective when a constant cost is assumed for all ages. This suggests that increases in costs by client age would not change the age prioritization substantially.

This analysis does not provide sufficient rationale for geographic targeting among provinces in South Africa. The model shows that the VMMC program is cost-saving in all provinces when using a unit cost of $125, although the uncertainty intervals include zero for the three provinces with the lowest projected HIV incidence—Western Cape, Limpopo, and Gauteng. This indicates that if future HIV incidence is on the low end of the projections in these three provinces, scaling up VMMC may not be cost-saving at this unit cost. Because the uncertainty around the HIV incidence estimates is too wide to rank the provinces by cost-effectiveness of the VMMC program, the country may instead wish to consider prioritizing resources among the provinces based on need for services, focusing most closely on provinces with the lowest VMMC coverage. These include Northern Cape (20.3% MC prevalence), KwaZulu-Natal (23.2%), Free State (30.0%), and North West (36.7%) [5]. The funding amount for each province will, of course, be proportional to the absolute size of the uncircumcised population, which is a function both of coverage and overall population size. Given policymakers’ desire to prioritize HIV prevention programs by geography, one recommendation would be to work toward obtaining better estimates of local incidence, because this is a key determinant of the impact of all HIV prevention technologies.

Model results should be considered in light of implementation trends. The country has seen that clients older than 25, and especially those older than 35, access VMMC services at lower rates than expected [10]. Because a strategy focusing on men ages 15–34 will maximize benefits of the program, increased investment in demand creation for men ages 25–34 should be piloted, and the relationship between the cost of the targeted demand creation and the number of clients in this age group accessing services should be determined. This cost relationship will determine the cost-effectiveness of focusing on this age group. Studies are under way in South Africa and elsewhere testing different approaches to increase demand among men ages 25–34.

Importantly, focusing VMMC efforts on specific age groups to ensure program efficiency and impact should not mean denying services to males outside these age groups. Model scenarios that include 10- to 14-year-olds show only small increases in HIV infections averted and result in a higher cost per HIV infection averted over 15 years. This is because males in this age group are generally not yet highly sexually active, so the impact of circumcising these males on HIV incidence will accordingly be delayed. However, 10- to 14-year-olds accounted for an estimated 42% of the VMMC client base in 2014, and the proportion of clients in this age group has increased since 2010 (S1 Table). Therefore, while circumcising males in this age group does not appear cost-effective in the short term, providing VMMC to those who seek services (without targeted demand creation for this group) will result in greater impact on HIV incidence in the long term (after 25 years). Once coverage targets in the upper age groups are achieved, it will also be necessary to continue circumcising adolescents to maintain this level of coverage over the long term. Thus, putting any policy in place to restrict age groups eligible for VMMC due to model results could harm the program’s ability to reach such groups in the future, as it will send a confusing message to communities. It should be mentioned that South African law requires informed consent for VMMC; upholding this is particularly important when circumcising minors.

### Limitations

The limitations of the DMPPT 2.0 model have been described in detail elsewhere [11], and are summarized here.

The model relies on available estimates of demographic and epidemiological data, and is subject to the biases and assumptions of these inputs (e.g., baseline MC prevalence, unit costs, and projections of future HIV incidence). Future HIV incidence, in particular, is dependent on many biological and behavioral influences and is therefore not possible to accurately project in the long term. Longer-term HIV incidence projections, which are key to estimating impact in the long term, are less certain than nearer-term projections.

The unit costs used in this paper were estimates advised by local stakeholders, roughly based on PEPFAR experience. After this analysis was conducted, a facility-based VMMC costing study was conducted in South Africa, the results of which are included in this manuscript collection [14]. The study found that the average unit cost of VMMC in South Africa is $132 USD, rather than the $125 used in this analysis. In addition, HIV treatment costs have decreased since these analyses were conducted. Therefore the projection that scaling up VMMC is cost-saving across all provinces except Gauteng may no longer hold.

Though the analysis attempts to take into account the escalating unit cost of VMMC by age, we were unable to obtain costing data on VMMC provision for clients of various age groups. To account for this, we employed a generous assumption in which the cost of providing services to older clients is twice as much as the cost of providing services to clients ages 10–19. While this assumption errs on the side of caution, the escalating unit-cost scenarios are based on estimates and not actual costing data.

Factors above and beyond age and geography hold significant implications for the implementation of the national VMMC program, such as stigma associated with VMMC and tensions over the practice of traditional circumcision [15,16]. Cultural norms around age of circumcision in some communities will also drive trends in client age in the VMMC program. When selecting a scale-up strategy or target, South Africa will also need to acknowledge other influences such as human resources, the political environment, and challenges to demand creation specific to certain subpopulations. Furthermore, because the model is not set up to reflect how costs may change over time as a program matures and becomes routine, program budgeting should be conducted through a separate process that accounts for the actual activities and commodities requiring funding through a specific funding stream.

## Conclusion

The DMPPT 2.0 exercise shows the relative cost-effectiveness and impact of circumcising different age groups of clients. As South Africa develops a national strategy for VMMC, the government may want to consider piloting increased demand creation focused on men ages 25–34, while still providing services to anyone coming in for services regardless of age. Because scaling up VMMC is projected to be cost-saving in all provinces, it will be advantageous for the country to continue scale-up everywhere, with a particular focus on provinces with low MC coverage.

## Supporting Information

S1 Appendix South Africa DMPPT 2.0 Model Inputs, Part 1. See Methods section for data sources.
(DOCX)

S2 Appendix South Africa DMPPT 2.0 Model Inputs, Part 2 (Epidemiology and Demographic Inputs). See Methods section for data sources.
(XLSX)

S1 Fig Provincial HIV incidence is inversely related to the number of VMMCs per HIV infection averted (VMMC/IA). (a) Average HIV incidence for each province, [2013 to 2028]; (b) Projected number of VMMCs per HIV infection averted for each province, [2013 to 2028]; IA = HIV infections averted.
(TIF)

S1 Table Estimated number of circumcisions conducted in South Africa, by age and year. Indicated percentage represents the portion of VMMCs done in this age group out of all ages in each year.
(DOCX)

S2 Table Number of VMMCs conducted in each South African province, by year. Indicated percentage represents the portion of VMMCs done in this province out of all provinces in each year.
(DOCX)
