# Correction: Cost and Impact of Voluntary Medical Male Circumcision in South Africa: Focusing the Program on Specific Age Groups and Provinces

**DOI:** 10.1371/journal.pone.0169802

**Published:** 2017-01-03

**Authors:** Katherine Kripke, Ping-An Chen, Andrea Vazzano, Ananthy Thambinayagam, Yogan Pillay, Dayanund Loykissoonlal, Collen Bonnecwe, Peter Barron, Eva Kiwango, Delivette Castor, Emmanuel Njeuhmeli

There is an error in the Funding statement. The number for the Cooperative Agreement Project SOAR (Supporting Operational AIDS Research) is incorrect. The correct number is OAA-A-14-00060.
