# Supplementary material for: Cost and Impact of Voluntary Medical Male Circumcision in South Africa: Focusing the Program on Specific Age Groups and Provinces
Source: PLoS One. 2016 Jul 13;11(7):e0157071. doi: 10.1371/journal.pone.0157071 (PMC4943592; doi:10.1371/journal.pone.0157071)
Supplement: S1 Appendix — See Methods section for data sources. (DOCX) [file pone.0157071.s001.docx]

| Input | Data | Reference |
| --- | --- | --- |
| Percent circumcised in base year, by 5-year age group (Provincial estimates below) | EIMC: 3.7%  5-9: 10.5%  10-14: 18.4%  15-19: 31.0%  20-24: 43.7%  25-29: 46.4%  30-34: 46.4%  35-39: 46.4%  40-44: 46.4%  45-49: 46.4%  50-54: 43.6%  55-59: 43.6% | South African National HIV Prevalence, Incidence and Behaviour Survey, 2012 (age 15-49)  Authors’ assumptions (EIMC, 5-9, 10-14, 50-54, 55-59) |
| Number of male circumcisions Performed, 2010-2013 | 2010: 140,000  2011: 348,000  2012: 443,000  2013: 415,000 | National program statistics |
| VMMC effectiveness | 0.6 | Auvert B, Taljaard D, Lagarde E, Sobngwi-Tambekou J, Sitta R, et al. (2005) Randomized, controlled intervention trial of male circumcision for reduction of HIV infection risk: The ANRS 1265 trial. PLoS Med 2(11): e298.  Gray RH, Kigozi G, Serwadda D, Makumbi F, Watya S, Nalugoda N et al. Male circumcision for HIV prevention n men in Rakai, Uganda: a randomized trial. Lancet 2007; 369: 767-66.  Bailey RC, Moses S, Parker CB, Agot K, Maclean I, Krieger JN, et al. Male circumcision for HIV prevention in young men in Kisumu, Kenya: a randomized controlled trial. Lancet 2007; 369: 643-56. |
| Ratio of infections averted among females to males | 0.49 | Spectrum, Goals Model |
| HIV incidence | See S2 Appendix – Spectrum Inputs | Authors’ Calculations  Spectrum, AIDS Impact Module and Goals module |
| Discount rate | 0.03 | Authors’ assumption. |

**Percent circumcised in base year, by 5-year age group (Provincial estimates)**

|  | Free State | Mpumalanga | North West | KwaZulu-Natal | Eastern Cape | Northern Cape | Western Cape | Limpopo | Gauteng |
| --- | --- | --- | --- | --- | --- | --- | --- | --- | --- |
| EIMC | 2.9% | 3.9% | 3.0% | 1.9% | 5.9% | 1.5% | 3.3% | 5.8% | 3.9% |
| 10-14 | 14.5% | 19.5% | 14.3% | 9.1% | 28.8% | 7.9% | 16.5% | 28.2% | 19.7% |
| 15-19 | 24.5% | 34.4% | 25.1% | 16.2% | 52.2% | 13.5% | 26.6% | 50.5% | 30.6% |
| 20-24 | 34.5% | 48.5% | 35.4% | 22.8% | 73.6% | 19.0% | 37.5% | 71.2% | 43.1% |
| 25-29 | 36.7% | 51.5% | 37.6% | 24.2% | 78.1% | 20.2% | 39.8% | 75.6% | 45.7% |
| 30-34 | 36.7% | 51.5% | 37.6% | 24.2% | 78.1% | 20.2% | 39.8% | 75.6% | 45.7% |
| 35-39 | 36.7% | 51.5% | 37.6% | 24.2% | 78.1% | 20.2% | 39.8% | 75.6% | 45.7% |
| 40-44 | 36.7% | 51.5% | 37.6% | 24.2% | 78.1% | 20.2% | 39.8% | 75.6% | 45.7% |
| 45-49 | 36.7% | 51.5% | 37.6% | 24.2% | 78.1% | 20.2% | 39.8% | 75.6% | 45.7% |
| 50-54 | 34.4% | 48.4% | 35.3% | 22.7% | 73.4% | 19.0% | 37.4% | 71.0% | 43.0% |
| 55-59 | 34.4% | 48.4% | 35.3% | 22.7% | 73.4% | 19.0% | 37.4% | 71.0% | 43.0% |
