# Supplementary figures and images for: Cost and Impact of Voluntary Medical Male Circumcision in South Africa: Focusing the Program on Specific Age Groups and Provinces
Source: PLoS One. 2016 Jul 13;11(7):e0157071. doi: 10.1371/journal.pone.0157071 (PMC4943592; doi:10.1371/journal.pone.0157071)

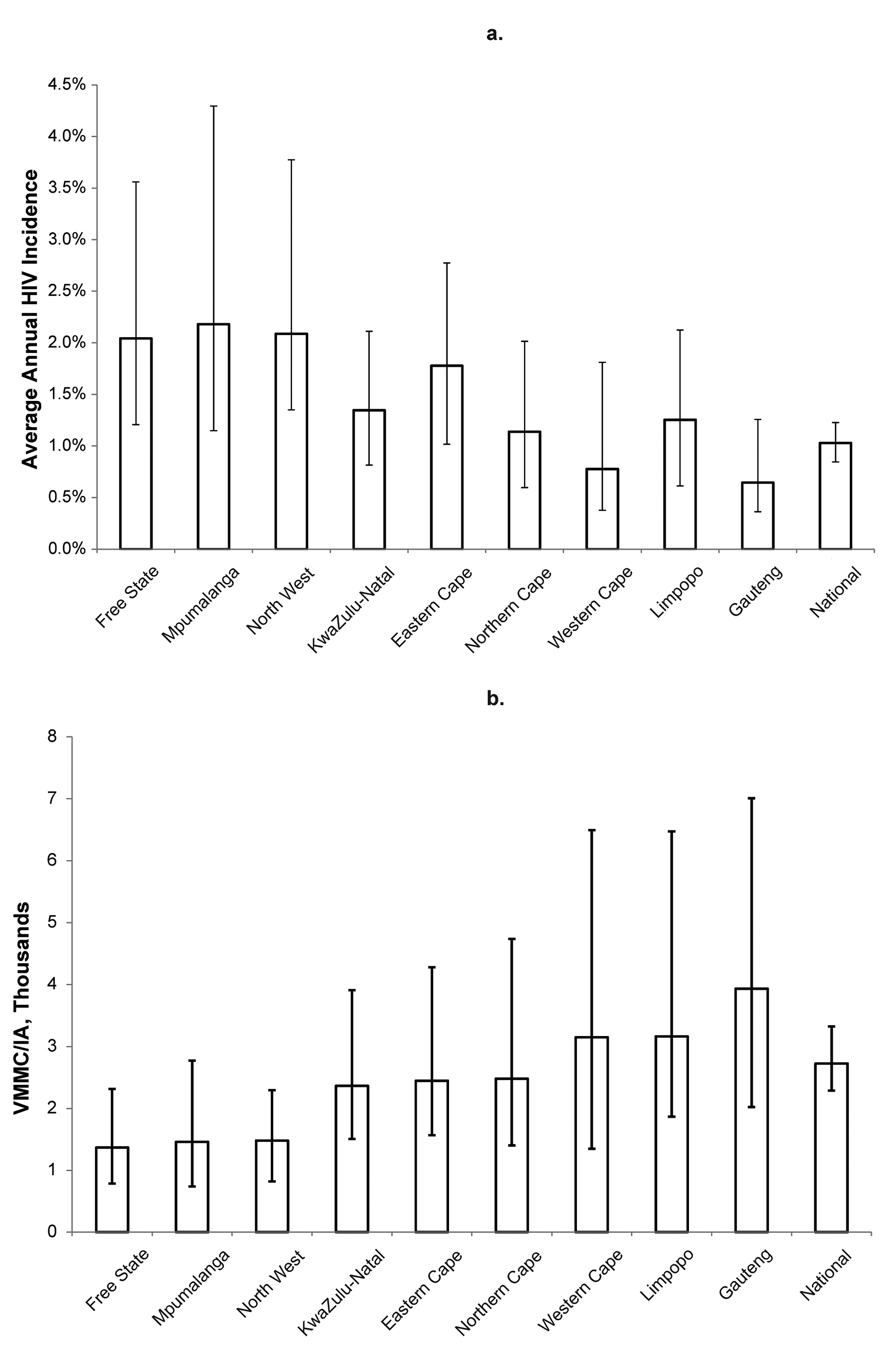

Supplement: S1 Fig — (a) Average HIV incidence for each province, [2013 to 2028]; (b) Projected number of VMMCs per HIV infection averted for each province, [2013 to 2028]; IA = HIV infections averted. (TIF) [file pone.0157071.s003.tif]
