# Supplementary material for: Cost and Impact of Voluntary Medical Male Circumcision in South Africa: Focusing the Program on Specific Age Groups and Provinces
Source: PLoS One. 2016 Jul 13;11(7):e0157071. doi: 10.1371/journal.pone.0157071 (PMC4943592; doi:10.1371/journal.pone.0157071)
Supplement: S1 Table — Indicated percentage represents the portion of VMMCs done in this age group out of all ages in each year. (DOCX) [file pone.0157071.s004.docx]

## S1 Table: Estimated number of circumcisions conducted in South Africa, by age and year. Indicated percentage represents the portion of VMMCs done in this age group out of all ages in each year.

| Age groups | 2010 | | 2011 | | 2012 | | 2013 | | 2014 | | All years | |
| --- | --- | --- | --- | --- | --- | --- | --- | --- | --- | --- | --- | --- |
| 10–14 | 2,498 | 41% | 9,087 | 41% | 47,389 | 29% | 283,034 | 41% | 327,144 | 45% | 669,153 | 41% |
| 15–19 | 1,607 | 27% | 5,845 | 27% | 54,152 | 33% | 177,259 | 26% | 172,853 | 24% | 411,716 | 25% |
| 20–24 | 759 | 13% | 2,760 | 13% | 28,432 | 17% | 91,383 | 13% | 88,203 | 12% | 211,537 | 13% |
| 25–34 | 803 | 13% | 2,919 | 13% | 25,193 | 15% | 97,337 | 14% | 98,260 | 13% | 224,512 | 14% |
| 35–49 | 319 | 5% | 1,161 | 5% | 8,880 | 5% | 36,776 | 5% | 38,142 | 5% | 85,278 | 5% |
| 50+ | 49 | 1% | 179 | 1% | 1,289 | 1% | 5,766 | 1% | 6,249 | 1% | 13,532 | 1% |
| Total | 6,035 | 100% | 21,951 | 100% | 165,335 | 100% | 691,555 | 100% | 730,851 | 100% | 1,615,728 | 100% |

**Methodology**: USAID and CDC circumcision data by month were provided by Clinton Health Access Initiative (CHAI), South Africa in 2015.CDC data was disaggregated by the age distribution of historical CDC data from all sites (fixed and mobile) provided by CHAI, using year-specific data (2010/2011 distribution was used for 2010/2011 data; 2011/12 distribution for 2011/2012, etc.). USAID data was disaggregated by the age distribution of historical USAID data from all sites (fixed and mobile) provided by CHAI, using year-specific data. Government-only data was derived by subtracting out the USAID and CDC numbers from the reported DHIS numbers, as provided by CHAI. Because the DHIS data only officially reports MCs in the 15-49 age group, the number of MCs in the 10-14 age group was estimated by applying the age distribution of historical USAID fixed-site circumcisions. The government-only data were then broken up further into age groups based again on the age distribution of historical USAID fixed-site circumcisions.
