# Supplementary material for: Cost and Impact of Voluntary Medical Male Circumcision in South Africa: Focusing the Program on Specific Age Groups and Provinces
Source: PLoS One. 2016 Jul 13;11(7):e0157071. doi: 10.1371/journal.pone.0157071 (PMC4943592; doi:10.1371/journal.pone.0157071)
Supplement: S2 Table — Indicated percentage represents the portion of VMMCs done in this province out of all provinces in each year. (DOCX) [file pone.0157071.s005.docx]

S2 Table: Number of VMMCs conducted in each South African province, by year. Indicated percentage represents the portion of VMMCs done in this province out of all provinces in each year.

| Province | 2010 | | 2011 | | 2012 | | 2013 | | 2014 | | All years | |
| --- | --- | --- | --- | --- | --- | --- | --- | --- | --- | --- | --- | --- |
| Eastern Cape | 0 | 0% | 0 | 0% | 203 | 0% | 10,279 | 1% | 15,144 | 2% | 25,626 | 2% |
| Free State | 0 | 0% | 206 | 1% | 5,901 | 4% | 28,594 | 4% | 37,757 | 5% | 72,458 | 4% |
| Gauteng | 0 | 0% | 1,422 | 6% | 63,902 | 39% | 241,345 | 35% | 210,319 | 29% | 516,988 | 32% |
| KwaZulu-Natal | 6,035 | 100% | 20,323 | 93% | 73,226 | 44% | 223,411 | 32% | 199,925 | 27% | 522,920 | 32% |
| Limpopo | 0 | 0% | 0 | 0% | 0 | 0% | 21,598 | 3% | 42,388 | 6% | 63,986 | 4% |
| Mpumalanga | 0 | 0% | 0 | 0% | 19,067 | 12% | 96,298 | 14% | 125,186 | 17% | 240,551 | 15% |
| North West | 0 | 0% | 0 | 0% | 0 | 0% | 40,950 | 6% | 59,897 | 8% | 100,847 | 6% |
| Northern Cape | 0 | 0% | 0 | 0% | 2,270 | 1% | 8,521 | 1% | 11,565 | 2% | 22,356 | 1% |
| Western Cape | 0 | 0% | 0 | 0% | 786 | 0% | 20,658 | 3% | 28,817 | 4% | 50,261 | 3% |
| Total | 6,035 |  | 21,951 |  | 165,353 |  | 691,654 |  | 730,998 |  | 1,615,993 |  |

**Methodology**: Provincial data for South Africa (males age 15-49, by South African fiscal year) were provided by Clinton Health Access Initiative, South Africa, in 2015.
